# Norepinephrine Reduces Reactive Oxygen Species (ROS) and DNA Damage in Ovarian Surface Epithelial Cells

**DOI:** 10.4172/1948-593X.1000127

**Published:** 2015-05-15

**Authors:** Pooja R Patel, Muralidhar L Hegde, Jacob Theruvathu, Sankar A Mitra, Istvan Boldogh, Lawrence Sowers

**Affiliations:** 1 Department of Obstetrics and Gynecology, The University of Texas Medical Branch in Galveston, Texas, USA; 2 Department of Radiation Oncology, Houston Methodist Research Institute, Houston, Texas, USA; 3 Department of Pharmacology and Toxicology, The University of Texas Medical Branch in Galveston, Texas, USA; 4 Department of Microbiology and Immunology, The University of Texas Medical Branch in Galveston, Texas, USA

## Abstract

**Objective:**

To determine the role of norepinephrine (NE) on DNA damage and reactive oxygen species (ROS) generation in ovarian surface epithelial cells.

**Method:**

Non-tumorigenic, immortalized ovarian surface epithelial cells were treated with NE, bleomycin, and bleomycin followed by NE. The comet assay was performed on each treatment group to determine the amount of single and double-strand breaks induced by treatments. ROS levels for each treatment group were measured using the H2DCF-DA fluorescence assay. Finally, RNA transcripts were measured for each treatment group with regards to the expression of DNA repair and oxidative stress genes.

**Results:**

The mean tail moment of untreated cells was significantly greater than that of cells treated with NE (p=0.02). The mean tail moment of cells treated with bleomycin was significantly greater than that of cells treated with bleomycin followed by NE (p<0.01). Treatment with NE resulted in significantly less ROS generation than in untreated cells (p<0.01). NE treatment after hydrogen peroxide treatment resulted in a noticeable decrease in ROS generation. Genes associated with oxidative stress were upregulated in cells treated with bleomycin, however this upregulation was blunted when bleomycin-treated cells were treated subsequently with NE.

**Conclusion:**

NE is associated with decreased DNA damage and ROS production in ovarian surface epithelial cells. This effect is protective in the presence of the oxidative-damaging agent bleomycin. These results suggest an additional physiologic role for the stress hormone NE, in protecting ovarian surface epithelial cells from oxidative stress.

## Introduction

Psychological stress is believed to be one of the significant factors involved in the development and progression of human cancer [1,2]. Stress has been shown to increase both tumor growth and invasiveness; however, the mechanisms underlying this phenomenon are as yet unresolved [3]. Sympathetic nervous system mediators, including the stress hormone norepinephrine (NE), might in part modulate this effect [4,5]. Sood et al. have shown that the adrenergic hormones epinephrine and NE increase the invasive potential of ovarian cancer cells in in vitro assays [3]. Some clinical studies investigating the potential role of β-adrenergic antagonists in cancer patients have shown that they increase patient survival [6], however, other studies investigating survival in patients with ovarian cancer could not confirm the efficacy of β-blocker treatment [7,8], suggesting that NE might be acting through alternative pathways.

The sympathetic innervation of the human ovary is critical for the regulation of multiple aspects of ovarian function including ovulation [9–12]. NE is the predominant catecholamine acting on β-adrenergic receptors in granulosa and thecal cells, stimulating steroidogenesis. In addition to responding to NE from sympathetic nerves, granulosa cells can synthesize and store NE as well as release it upon depolarization. Much less is known about the effects of NE on the ovarian surface epithelium (OSE), the third cell type found in the mammalian ovary.

The OSE constitutes the outer layer of the ovary, undergoing rupture and repair with each ovulatory cycle [13]. Reactive oxygen species (ROS) are involved in follicular rupture at the ovarian surface during ovulation [14–16]. Collateral damage from cyclic ROS generation occurs to the deoxyribonucleic acid (DNA) of the OSE which requires repair prior to proliferation. Most ovarian cancers are thought to arise from undifferentiated cells in the OSE and therefore ROS-mediated damage to stem-like cells within the OSE could be a critical factor in ovarian cancer etiology.

The catecholamines (dopamine, epinephrine, NE) are known primarily as neurotransmitters in the central and peripheral nervous systems; however, they can easily undergo oxidation forming a complex array of products [17]. Two scenarios can result from these reactions. On the one hand, catecholamines can act as antixoidants, scavenging both singlet oxygen and superoxide and protecting DNA from ROS-mediated DNA cleavage [18,19]. Alternatively, reaction products formed by oxidation of the catecholamines including unstable quinones and adrenochromes, can damage DNA [20–22] and result in cell damage. Research shows that NE can be either protective or damaging to mammalian cells, depending upon the cell type and NE concentration. In granulosa cells, NE at 10 nM levels increased ROS levels, but this effect was considered to be a normal physiologic event that did not reduce cell viability and was independent of the β-adrenergic receptor [23]. Rather, an active NE transporter (NET) has been identified which allows for cellular uptake of NE into the cellular cytoplasm, bypassing the β-adrenergic receptor mediated signaling cascade and implying a receptor-independent role of NE [11]. Low levels of NE (0.3–10 μM) have been shown to protect dopaminergic neurons [24] independently of β--receptor activation, perhaps by acting as an antioxidant or metal chelator [25]. In contrast, NE acting through α-adrenergic receptors increased superoxide production in primary human peripheral blood mononuclear cells, suggesting the physiologic role of NE might be cell-type specific [26].

The relationship between ROS generation and NE has not been previously examined in OSE cells. In this study, we investigated the effect of NE on the hTERT-immortalized cell line IOSE-29. DNA damage was assessed by the comet assay in the presence and absence of the DNA-damaging agent, bleomycin. Levels of intracellular ROS in the presence and absence of exogenous hydrogen peroxide were also measured. The effect of NE on the transcriptional level of a battery of genes involved in attenuating ROS and repairing DNA was also assessed.

## Materials and Methods

### Cell line generation and culture methods

Transfection of human ovarian surface epithelial cells with the SV40 early region expressing T/t antigen and then subsequent infection with a retrovirus containing a full-length hTERT cDNA, to create the IOSE-29 (Immortalized Ovarian Surface Epithelial-29) cell line has been previously described [27,28]. For this study, IOSE-29 cells were cultured in 10 cm dishes with 10 ml of ovarian epithelial-cell culture medium consisting of 1:1 MCDB105 and Media 199 (Sigma-Aldrich) supplemented with 10% fetal bovine serum and 1% penicillin/ streptomycin (Life Technologies, Inc.).

### Cell treatments

#### Control cells

Cells were incubated with serum free media for 24 hours prior to and for the duration of experiments. All cells were detached simultaneously using serum free media and a cell scraper.

#### Treatment with NE

Cells were incubated with serum free media for 24 hours prior to experiments. Cells were incubated with 10^−5^ M NE (Sigma-Aldrich, N5785) in serum-free media for 30 min at 37°C. Previous studies have shown that NE does not result in considerable changes in DNA integrity until 10 min [29]. We therefore chose 30 minutes as the time period to treat cells. We chose 10^−5^ M as this is the concentration in the serum during physiologic stress [30]. Cells were then washed twice (5 min each) with 1x PBS and incubated with serum-free media at 37°C for the duration of the experiment. All cells were detached simultaneously using serum free media and a cell scraper.

#### Treatment with Bleomycin

Cells were incubated with 2.5 ug/ml bleomycin (15 unit bottle, TEVA Parenteral Medicines Inc, 92618) in serum-free media for 30 minutes at 37°C to induce single and double strand DNA breaks. Cells were then washed twice (5 min each) with 1x PBS and incubated with serum-free media at 37°C for the duration of the experiment. All cells were detached simultaneously using serum free media and a cell scraper.

#### Treatement with bleomycin followed by NE

Cells were incubated with 2.5 ug/ml bleomycin in serum-free media for 30 min at 37°C. Cells were then washed twice (5 min each) with 1x PBS and then incubated with 10^−5^ M NE (Sigma-Aldrich, N5785) in serum-free media for 30 minutes at 37°C. Cells were then washed twice (5 min each) with 1x PBS. All cells were detached simultaneously using serum free media and a cell scraper.

### Cell viability assay

A cell viability assay was done to determine whether treatment with NE would alter viability of the cells, as this would affect interpretation of results from DNA damage and PCR expression assays. Approximately 5 × 10^4^ cells were plated in 10 cm cell culture dishes. Treated cells were incubated with 10^−5^ M NE for 30 min. At 24 and 48 h, cells were manually counted in triplicate samples.

### Comet assay to assess DNA damage

The Trevigen alkaline comet assay kit (4250-050-k) was used to measure DNA damage in each sample. Cells were treated, isolated and mixed in low melting point agarose in PBS and pipetted onto slides supplied by the kit. After the gels were allowed to set at 4°C, the slides were immersed in lysis buffer (4250-050-01, Trevigen) for 40 min. After excess buffer was drained from the slides, the slides were immersed in Alkaline Unwinding Solution (200 mM NaOH, 1mM EDTA) for 20 min at room temperature. The slides were then placed in an electrophoresis tray, submerged in alkaline electrophoresis solution (200 mM NaOH, 1mM EDTA, pH 8) with the power supply set at 21 volts for 30 min. Excess electrophoresis solution was drained, and slides were gently immersed twice in dH_2_O for 5 min each, then in 70% ethanol for 5 min. Samples were dried for 30 min at 37°C. SYBR Gold solution was placed onto the dried slides and removed after 30 minutes. Slides were dried and submitted to our university core facility lab for evaluation by epifluoresecence microscopy. Of note, the personnel from the core facility lab were unaware of the sample source, adding an extra layer of blinding to the results.

Mean tail moment was used to measure DNA break frequency and, thus, DNA damage. The mean tail moment was obtained by selecting at random one hundred comets per sample and averaging the tail moment for this sample. Tail moment was calculated by multiplying the length of the tail by the percent DNA in the tail. Each experiment was performed in triplicate.

### ROS quantification

Changes in intracellular ROS levels were determined using the fluorogenic probe 5-(and-6)-chloromethyl-2′,7′-dichlorodihydrofluorescein diacetate acetyl ester (CM-H2DCF-DA; Invitrogen, Eugene, OR, USA). Briefly, cells were grown to 70% confluence and loaded with 50 μM CM-H2DCF-DA at 37°C for 30 min. Cells were then washed with PBS. Cells were treated with 40 uM Hydrogen peroxide (H_2_O_2_) (Fisher, Fair Lawn, NJ, USA) as a positive control. Empty wells without plated cells were used as a negative control. Changes in DCF fluorescence were recorded on an FLx800 (Bio-Tek Instruments, Winooski, VT, USA) microplate reader at 485 nm excitation and 528 nm emission, at 10 min intervals. For the H_2_O_2_→NE samples, cells were treated with 40 μM H_2_O_2_ and fluorescence measurements were obtained at 10 min intervals. After 30 min, 10 μM NE was added to the wells and fluorescence measurements were continued at 10 min intervals. Results are expressed as fold change or arbitrarily in fluorescence units (FU). Experiments were performed in triplicate.

### RNA extraction and quantification

Total cellular RNA was extracted from cells using RNAqueous^™^ total RNA isolation kits (Life Technologies, CA) according to the manufacture’s recommendations. Subsequent to extraction, RNA was quantitated spectrophotometrically using a NanoDrop ND-1000 (NanoDrop Techniologies, DE). The quality of the purified RNA was assessed by visualization of 18S and 28S RNA bands using an Agilent BioAnalyzer 2100 (Agilent Technologies, CA). Resulting electropherograms were used in the calculation of the 28S/18S ratio and the RNA Integrity Number [31].

### Real time SYBR green QPCR gene expression analysis

The DNA Repair PCR Array (PAHS-042ZF-2 Qiagen) and the Human Oxidative Stress PCR Array (PAHS-065z Qiagen) was used as the template for genes transcribed following oxidative stress. Reverse transcription was performed on 0.5 mg of total RNA, utilizing RT^2^ First Strand Kit (Qiagen) as recommended by the manufacturer. The resulting cDNA was used as template for the subsequent PCR reaction, consisting of 2X RT^2^ SYBR Green Master Mix, template cDNA and reagent grade H_2_O in a total volume of 2700 ul. A multi-channel pipette was use to distribute 25 ul of the reaction mix to each well of the 96 well plate. Thermal cycling was carried out with a Roche LightCycler 480 II (Roche, USA) per manufacture’s recommendations (95°C, 10 min; and 40 cycles at 95°C, 15 S; 60°C, 1 min). Each experiment was performed in triplicate.

### Statistical analysis

Statistical significance of observed differences was defined as a p<0.05. Paired t-tests were used to calculate statistical differences, pairing results within experiments. All experiments were performed in triplicate. Calculations were done in R (R Foundation for Statistical Computing, 2014).

## Results

### Cell viability assay

**Figure 1:**
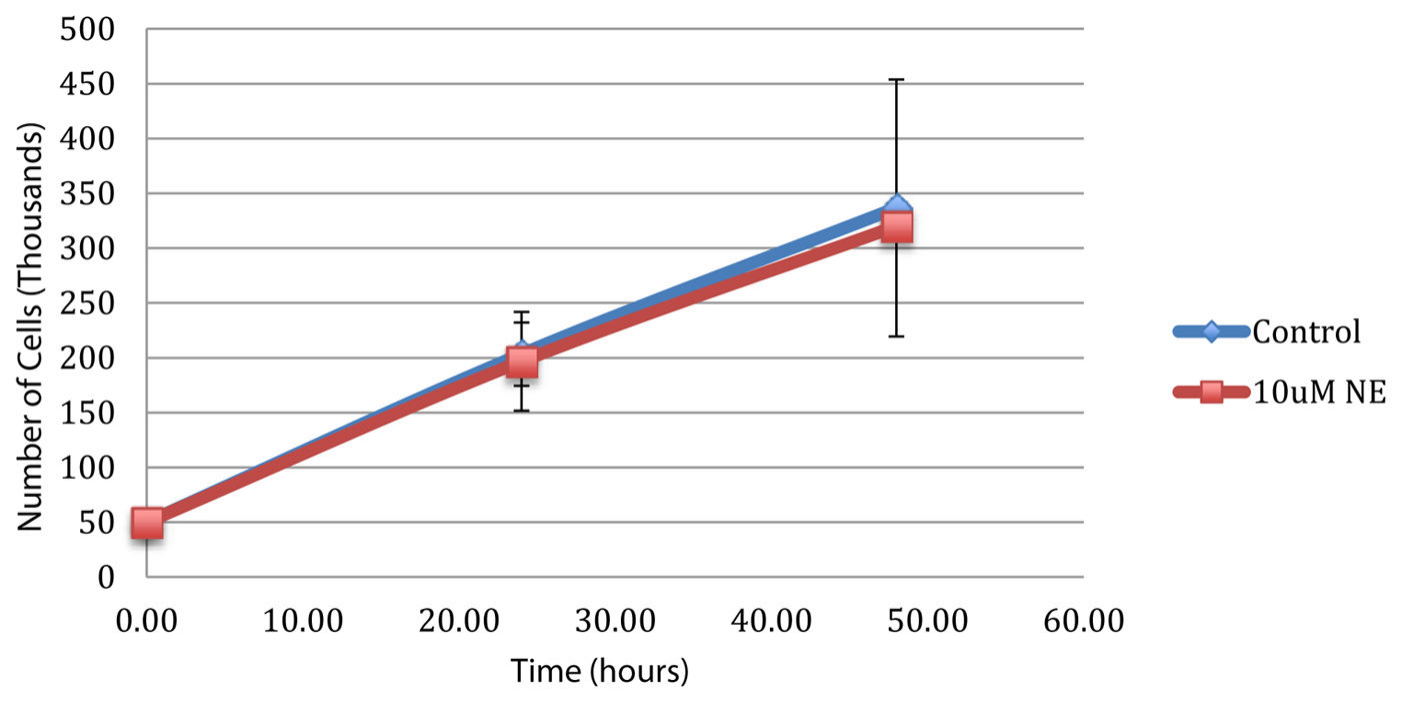
Cell viability assay. Presence of 10 uM norepinephrine does not affect cell viability of IOSE-29 ovarian surface epithelial cells. NE: Norepinephrine.

Cell viability was similar with and without treatment with NE (Figure 1).

### NE resulted in decreased DNA damage in ovarian surface epithelial cells

**Figure 2:**
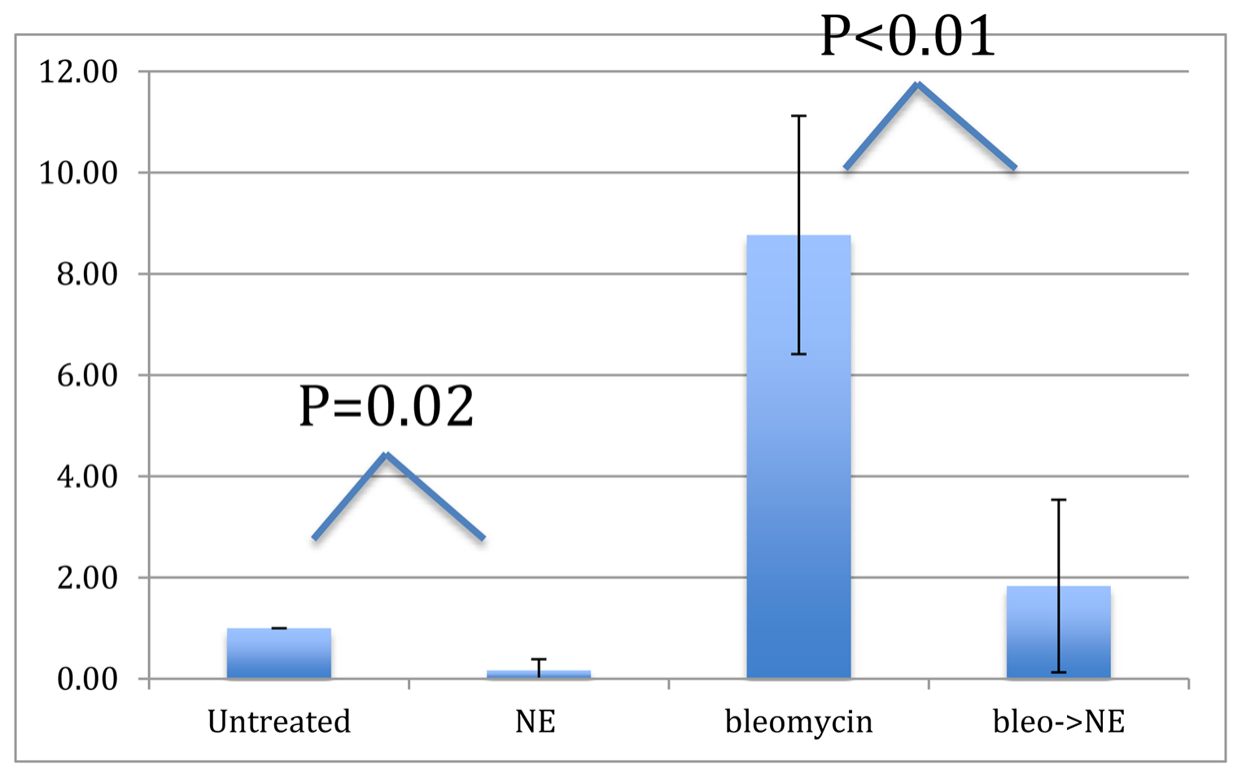
Comet assay results. This graph represents the levels of DNA damage with respect to untreated ovarian surface epithelial cells as a reference. DNA damage in cells treated with norepinephrine is significantly less than DNA damage in untreated cells (p=0.02). DNA damage in cells treated with bleomycin followed by norepinephrine is significantly less than DNA damage in cells treated with only bleomycin (p=0.01). NE: Norepinephrine; Bleo: Bleomycin; Bleo->NE: 30 minute treatment with bleomycin followed by 30 minute treatment with norepinephrine.

**Figure 3:**
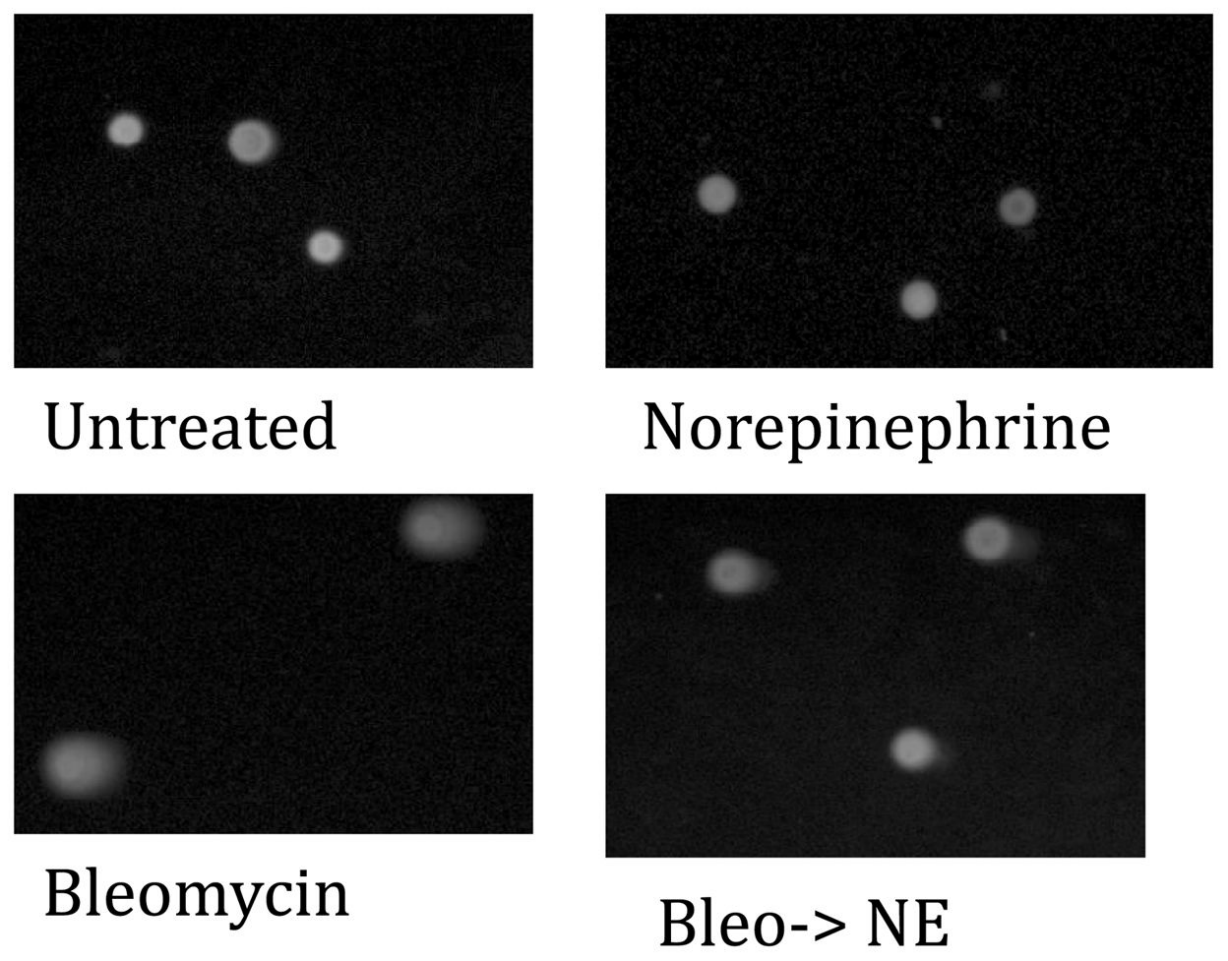
Comet assay images. Images of comet assay results of IOSE-29 cells. As expected, treatment with bleomycin resulted in larger comet tails, signifying greater DNA damage. Treatment with norepinephrine resulted in less tail, and subsequent treatment with norepinephrine after treatment with DNA damaging agent resulted in smaller tail. Bleo: Bleomycin; NE: Norepinephrine; Bleo->NE: 30 minute treatment with bleomycin followed by 30 minute treatment with norepinephrine.

DNA damage was assessed via mean tail moment, which represents the relative amount of damaged DNA present in the comet tail. Comet assay data is summarized in Figures 2 and 3. The mean tail moment of the untreated ovarian surface epithelial cells was 2.40 ± 0.47 arbitrary units, while the mean tail moment after treatment with bleomycin was 20.80 ± 6.1, signifying greater DNA damage. Treatment with NE resulted in a mean tail moment of 0.25 ± 0.19, a 10-fold decrease from untreated values (p=0.02). Treatment with bleomycin followed by treatment with NE resulted in a mean tail moment of 3.48 ± 0.90, a 6-fold decrease from the mean tail moment of treatment with bleomycin alone (p<0.01).

### NE resulted in decreased levels of ROS

**Figure 4:**
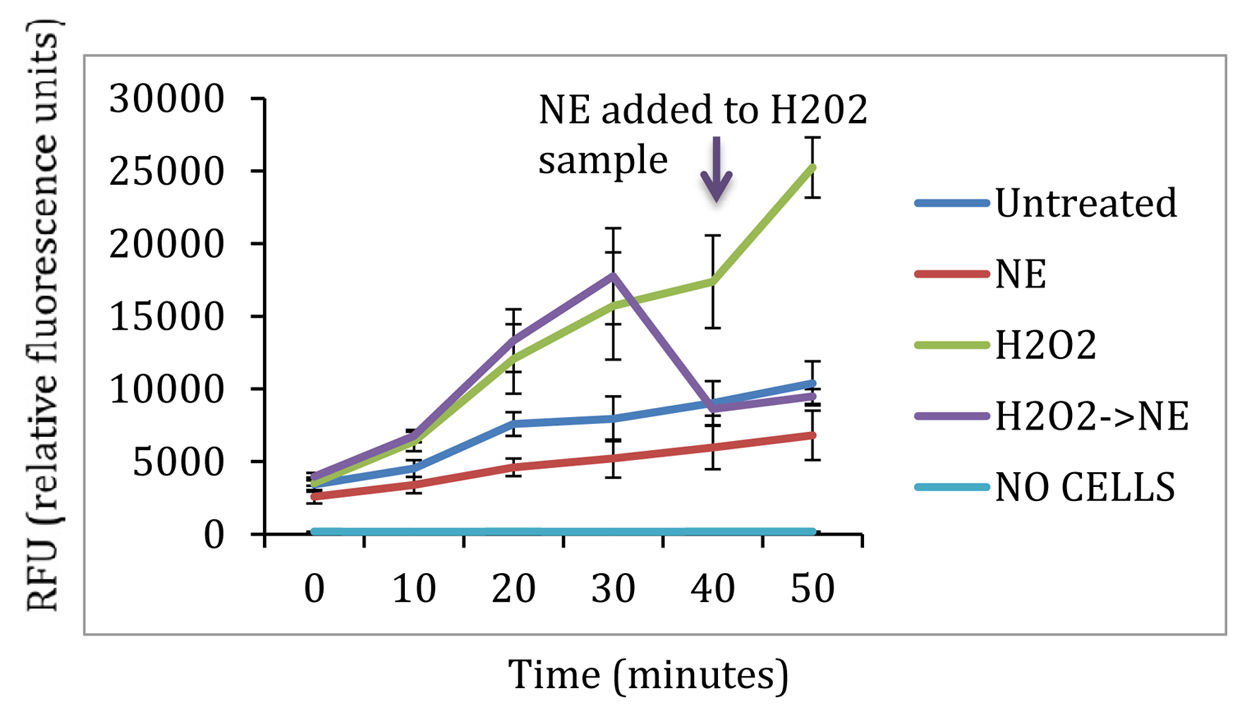
ROS generation measured by H2DCFDA. As expected, treatment with hydrogen peroxide resulted in greater ROS generation. Treatment with norepinephrine resulted in significantly less ROS generation than in untreated cells (p<0.01). Norepinephrine treatment after hydrogen peroxide treatment resulted in decreased ROS generation, however this different was not significant (p=0.14). H_2_O_2_: Hydrogen Peroxide; NE: Norepinephrine.

ROS data is summarized in Figure 4. Treatment with NE resulted in decreased ROS, as exhibited by decreased fluorescence emission throughout the 50 min experimental period. In addition, when comparing the treatment with H_2_O_2_ with the treatment with H_2_O_2_ followed by NE, both groups initially had similar fluorescence patterns until the NE was added to the second group, resulting in an abrupt decrease in fluorescence. There was significantly less ROS generation when cells were treated with NE compared to untreated cells (p<0.01). There tended to be less ROS generation after treatment with H_2_O_2_ followed by NE compared to treatment with only H_2_O_2_ (p=0.14).

### NE resulted in a decreased expression of oxidative stress-associated genes

**Figure 5:**
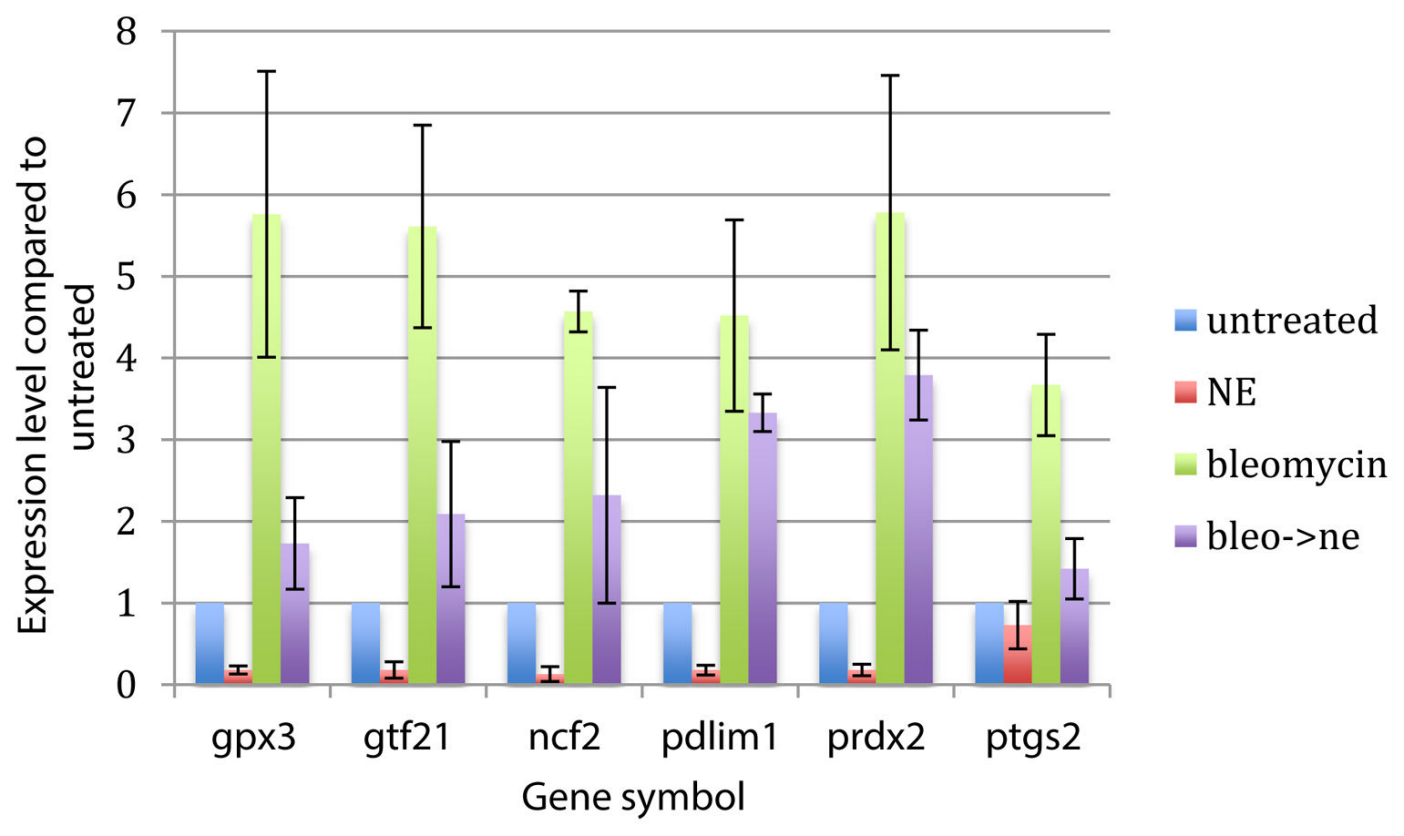
mRNA expression level of oxidative stress associated genes. All results are normalized to untreated cells. As expected, treatment with bleomycin resulted in increased expression of these genes when compared to untreated cells. Treatment with norepinephrine resulted in decreased expression of these genes when compared to untreated cells and treatment with norepinephrine after treatment with bleomycin resulted in decreased expression of these genes when compared with bleomycin treatment alone. Bleo: Bleomycin; NE: Norepinephrine; Bleo->NE: treatement with bleomycin for 30 minutes followed by treatment with norepinephrine for 30 minutes.

All oxidative stress genes with significant changes in transcription after treatment with norepinephrine showed decreased transcription after treatment with norepinephrine compared to the control. The same was observed with bleomycin followed by norepinephrine treatment compared to bleomycin treatment only. The specific fold changes are summarized in Figure 5.

## Discussion

Our results suggest that NE has a protective effect against oxidative stress in IOSE-29 cells, since treatment with NE resulted in reduced production of ROS and reduced DNA damage. Studies have shown that excess NE, generated by stress, might enhance the development and progression of ovarian cancer [3]. Our results thus provide a mechanistic connection between NE levels and ovarian cancer.

Baseline serum levels of NE have been reported to be in the 2–3 nM range rising by a factor of 4 under physiological stress [23,32,33]. Higher levels of NE are found in human preovulatory follicles, where concentrations as high as high as 45 nM have been reported [23]. In a previous study, Sood et al. demonstrated that the invasiveness of cultured ovarian cancer cells increased with increasing NE concentration up to 10 μM [23]. We therefore used this concentration in our experiments. Further studies are warranted to determine how lower concentrations of NE (i.e. physiologic concentrations) affect DNA integrity.

**Figure 6:**
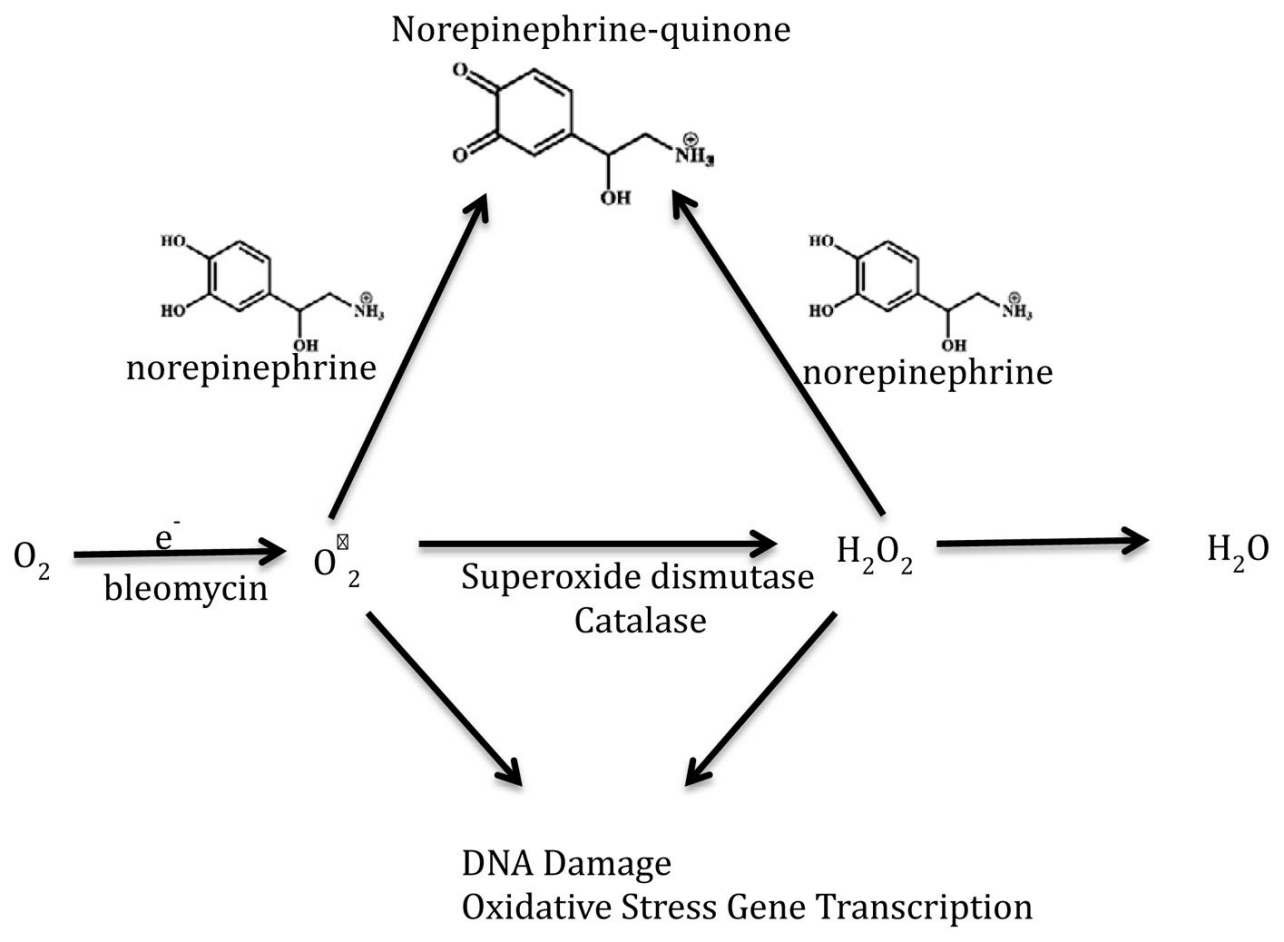
Proposed model of norepinephrine’s antioxidant effect in ovarian surface epithelial cells. Outlined above is the proposed pathway by which norepinephrine decreases ROS generation and subsequent DNA damage in ovarian surface epithelial cells. In this model, norepinephrine acts as a rapid ROS scavenger leading to a decrease in downstream DNA damage and reactive transcription of genes involved in the cellular response to oxidative stress.

Our results establish that in IOSE-29 cells in culture, added NE does not diminish cell viability, but reduces levels of DNA damage and ROS levels under standard culture conditions as well as in response to ROS-generating molecules including H_2_O_2_ and bleomycin. The time-dependence of these effects, as well as the effect of NE on gene expression levels, suggest that NE is acting directly as an antioxidant in these cells under these experimental conditions. Upon the basis of these findings, we propose a pathway by which NE acts as a rapid ROS scavenger. NE causes a decrease in ROS, resulting in a decrease in downstream DNA damage and decreased transcription of genes involved in the cellular response to oxidative stress and antioxidant defense (Figure 6).

The implications of these findings on normal ovarian physiology are of interest. Cyclic increases in NE during ovulation could serve to protect the DNA of OSE stem cells from damage that otherwise might result in genetic mutations. On the other hand, normal or stress-related higher levels of NE could also enhance the survival of tumorigenic cells, especially in response to DNA-damaging chemotherapy. The capacity of NE to enhance cell survival by non-adrenergic mechanisms might explain why β-blockers do not consistently improve survival in ovarian cancer patients.

Our results might also be of significance within the context of polycystic ovary syndrome (PCOS). In PCOS, hyperactivation of the sympathetic innervation of the ovary results in ovarian cyst formation and ovulatory failure [10]. Enhanced NE release would be expected to result in increased NE in the follicular fluid of patients with PCO. Paradoxically, both follicular fluid and granulosa cells from PCOS women have significantly lower NE levels [23]. Women with PCOS also have substantially higher levels of markers for oxidative stress and decreased antioxidant status [34,35]. Perhaps increased ROS in PCOS women cause the diminished NE levels. Diminished NE levels would result in less protection of DNA in OSE stem cells and increased mutation potential, perhaps explaining in part the association between PCOS and ovarian cancer [36]. In support of this mechanism, Macarthur et al. have shown that increased free radical production in septic stress is sufficient to oxidize and deactivate catecholamines, including exogenous catecholamines [37]. A superoxide dismutase small molecule mimetic restored vasopressor responses to NE. Perhaps such agents would be of value in treating PCOS.

In summary, we observe that NE decreases ROS in IOSE-29 cells in culture and reduces DNA damage. This effect is likely mediated by a direct radical scavenging property of NE as opposed to activation of adrenergic receptors. Cyclic release of NE in the ovary at the time of ovulation could protect replicating cells, including stem cells, from promutagenic insults, decreasing ovarian cancer risk. Increased local or systemic inflammation could result in diminished NE levels seen in PCOS which might be related to ovulatory failure and increased ovarian cancer risk.

## References

[1] Yuan A, Wang S, Li Z, Huang C (2010). Psychological aspect of cancer: From stressor to cancer progression. Exp Ther Med.

[2] Antoni MH, Lutgendorf SK, Cole SW, Dhabhar FS, Sephton SE (2006). The influence of bio-behavioural factors on tumour biology: pathways and mechanisms. Nat Rev Cancer.

[3] Sood AK, Bhatty R, Kamat AA, Landen CN, Han L (2006). Stress hormone-mediated invasion of ovarian cancer cells. Clin Cancer Res.

[4] Lutgendorf SK, Cole S, Costanzo E, Bradley S, Coffin J (2003). Stress-related mediators stimulate vascular endothelial growth factor secretion by two ovarian cancer cell lines. Clin Cancer Res.

[5] Toufexis D, Rivarola MA, Lara H, Viau V (2014). Stress and the reproductive axis. J Neuroendocrinol.

[6] Diaz ES, Karlan BY, Li AJ (2012). Impact of beta blockers on epithelial ovarian cancer survival. Gynecol Oncol.

[7] Heitz F, du Bois A, Harter P, Lubbe D, Kurzeder C (2013). Impact of beta blocker medication in patients with platinum sensitive recurrent ovarian cancer-a combined analysis of 2 prospective multicenter trials by the AGO Study Group, NCIC-CTG and EORTC-GCG. Gynecol Oncol.

[8] Johannesdottir SA, Schmidt M, Phillips G, Glaser R, Yang EV (2013). Use of β-blockers and mortality following ovarian cancer diagnosis: a population-based cohort study. BMC Cancer.

[9] Lara HE, Porcile A, Espinoza J, Romero C, Luza SM (2001). Release of norepinephrine from human ovary: coupling to steroidogenic response. Endocrine.

[10] Lara HE, Dorfman M, Venegas M, Luza SM, Luna SL (2002). Changes in sympathetic nerve activity of the mammalian ovary during a normal estrous cycle and in polycystic ovary syndrome: Studies on norepinephrine release. Microsc Res Tech.

[11] Greiner M, Paredes A, Rey-Ares V, Saller S, Mayerhofer A (2008). Catecholamine uptake, storage, and regulated release by ovarian granulosa cells. Endocrinology.

[12] Ricu M, Paredes A, Greiner M, Ojeda SR, Lara HE (2008). Functional development of the ovarian noradrenergic innervation. Endocrinology.

[13] Wong AS, Leung PC (2007). Role of endocrine and growth factors on the ovarian surface epithelium. J Obstet Gynaecol Res.

[14] Murdoch WJ, Townsend RS, McDonnel AC (2001). Ovulation-induced DNA damage in ovarian surface epithelial cells of ewes: prospective regulatory mechanisms of repair/survival and apoptosis. Biol Reprod.

[15] Murdoch WJ, Martinchick JF (2004). Oxidative damage to DNA of ovarian surface epithelial cells affected by ovulation: carcinogenic implication and chemoprevention. Exp Biol Med.

[16] Shkolnik K, Tadmor A, Ben-Dor S, Nevo N, Galiani D (2011). Reactive oxygen species are indispensable in ovulation. Proc Natl Acad Sci U S A.

[17] Bindoli A, Rigobello MP, Deeble DJ (1992). Biochemical and toxicological properties of the oxidation products of catecholamines. Free Radic Biol Med.

[18] Gülçin I1 (2009). Antioxidant activity of L-adrenaline: a structure-activity insight. Chem Biol Interact.

[19] Kawashima T, Ohkubo K, Fukuzumi S (2010). Radical scavenging reactivity of catecholamine neurotransmitters and the inhibition effect for DNA cleavage. J Phys Chem B.

[20] Djelic N, Anderson D (2003). The effect of the antioxidant catalase on oestrogens, triiodothyronine, and noradrenaline in the Comet assay. Teratog Carcinog Mutagen Suppl.

[21] Neri M, Cerretani D, Fiaschi AI, Laghi PF, Lazzerini PE (2007). Correlation between cardiac oxidative stress and myocardial pathology due to acute and chronic norepinephrine administration in rats. J Cell Mol Med.

[22] Spencer WA, Jeyabalan J, Kichambre S, Gupta RC (2011). Oxidatively generated DNA damage after Cu(II) catalysis of dopamine and related catecholamine neurotransmitters and neurotoxins: Role of reactive oxygen species. Free Radic Biol Med.

[23] Saller S, Merz-Lange J, Raffael S, Hecht S, Pavlik R (2012). Norepinephrine, active norepinephrine transporter, and norepinephrine-metabolism are involved in the generation of reactive oxygen species in human ovarian granulosa cells. Endocrinology.

[24] Troadec JD, Marien M, Darios F, Hartmann A, Ruberg M (2001). Noradrenaline provides long-term protection to dopaminergic neurons by reducing oxidative stress. J Neurochem.

[25] Paris I, Martinez-Alvarado P, Perez-Pastene C, Vieira MN, Olea-Azar C (2005). Monoamine transporter inhibitors and norepinephrine reduce dopamine-dependent iron toxicity in cells derived from the substantia nigra. J Neurochem.

[26] Deo SH, Jenkins NT, Padilla J, Parrish AR, Fadel PJ (2013). Norepinephrine increases NADPH oxidase-derived superoxide in human peripheral blood mononuclear cells via alpha-adrenergic receptors. Am J Physiol Regul Integr Comp Physiol.

[27] Maines-Bandiera SL, Kruk PA, Auersperg N (1992). Simian virus 40-transformed human ovarian surface epithelial cells escape normal growth controls but retain morphogenetic responses to extracellular matrix. Am J Obstet Gynecol.

[28] Liu J, Yang G, Thompson-Lanza JA, Glassman A, Hayes K (2004). A genetically defined model for human ovarian cancer. Cancer Res.

[29] Flint MS, Baum A, Chambers WH, Jenkins FJ (2007). Induction of DNA damage, alteration of DNA repair and transcriptional activation by stress hormones. Psychoneuroendocrinology.

[30] Rupprecht M, Salzer B, Raum B, Hornstein OP, Koch HU (1997). Physical stress-induced secretion of adrenal and pituitary hormones in patients with atopic eczema compared with normal controls. Exp Clin Endocrinol Diabetes.

[31] Schroeder A, Mueller O, Stocker S, Salowsky R, Leiber M (2006). The RIN: an RNA integrity number for assigning integrity values to RNA measurements. BMC Mol Biol.

[32] Jiménez-Jiménez M, García-Escalona A, Martín-López A, De Vera-Vera R, De Haro J (2013). Intraoperative stress and anxiety reduction with music therapy: a controlled randomized clinical trial of efficacy and safety. J Vasc Nurs.

[33] Strahler J, Fischer S, Nater UM, Ehlert U, Gaab J (2013). Norepinephrine and epinephrine responses to physiological and pharmacological stimulation in chronic fatigue syndrome. Biol Psychol.

[34] Sabuncu T, Vural H, Harma M, Harma M (2001). Oxidative stress in polycystic ovary syndrome and its contribution to the risk of cardiovascular disease. Clin Biochem.

[35] Murri M, Luque-Ramírez M, Insenser M, Ojeda-Ojeda M, Escobar-Morreale HF (2013). Circulating markers of oxidative stress and polycystic ovary syndrome (PCOS): a systematic review and meta-analysis. Hum Reprod Update.

[36] Barry JA, Azizia MM, Hardiman PJ (2014). Risk of endometrial, ovarian and breast cancer in women with polycystic ovary syndrome: a systematic review and meta-analysis. Hum Reprod Update.

[37] Macarthur H, Westfall TC, Riley DP, Misko TP, Salvemini D (2000). Inactivation of catecholamines by superoxide gives new insights on the pathogenesis of septic shock. Proc Natl Acad Sci U S A.

